# Systemic therapy outcomes following separation surgery versus maximal feasible resection for spinal metastases from non-small cell lung cancer: a real-world retrospective cohort study

**DOI:** 10.1186/s12957-026-04300-y

**Published:** 2026-03-11

**Authors:** Zhilong Shen, Pengru Wang, Hao Yuan, Bo Li, Jianru Xiao, Wei Xu

**Affiliations:** Department of Orthopedic Oncology, Changzheng Hospital, Naval Military Medical University, 415 Fengyang Road, Shanghai, 200003 China

**Keywords:** Spinal metastases, Separation surgery, Maximal feasible resection, Systemic therapy

## Abstract

**Study design:**

Retrospective cohort study

**Objective:**

To compare systemic therapy outcomes between separation surgery and maximal feasible resection for patients with spinal metastases from non-small cell lung cancer (NSCLC).

**Summary of background data:**

The presence of bone metastases has been associated with diminished response of primary tumors to systemic therapy, especially immunotherapy. However, in patients with spinal metastases, the impact of varying degrees of bone metastatic burden reduction on the efficacy of subsequent systemic therapy remains unknown.

**Materials and methods:**

Adult patients (≥18 years) with spinal metastases from NSCLC who underwent surgical intervention at our institution between between January 2019 and June 2024 were retrospectively identified. Patients were categorized into two groups based on surgical strategy: separation surgery (SS) or maximal feasible resection (MFR). Systemic therapy response (objective response rate, disease control rate and best overall response) was assessed by Response Evaluation Criteria in Solid Tumors (RECIST) version 1.1. Surgical outcomes included blood loss, operating time, intraoperative blood transfusion, and complications. Overall survival (OS) was analyzed using the Kaplan–Meier method, and differences between groups were compared using the log-rank test.

**Results:**

A total of 36 patients with spinal metastases from NSCLC were included. Patients in the MFR group exhibited a significantly higher disease control rate compared to those in the SS group (71.4% vs. 27.3%, P = 0.024). However, MFR was associated with increased surgical morbidity, including greater intraoperative blood loss [median 800 mL (IQR: 650–1425) vs. 500 mL (IQR: 100–1000), P = 0.041], a higher rate of intraoperative blood transfusion (71.4% vs. 31.8%, P = 0.048), and more intraoperative complications (28.6% vs. 0%, P = 0.017). There was no statistically significant difference in overall survival between the two surgical strategies [median 23.7 months (95% CI: 18.8–29.4) vs. 20.6 months (95% CI: 17.5–22.5); HR = 0.42 (95% CI: 0.16–1.08); P = 0.065]. However, Exploratory subgroup analyses indicated that MFR combined with denosumab was associated with a significantly lower hazard of death compared with SS plus bisphosphonates (HR = 0.14, 95% CI: 0.03–0.61, P = 0.009) and MFR plus bisphosphonates (HR = 0.15, 95% CI: 0.02–0.95, P = 0.044). A non-significant trend was also observed versus SS combined with denosumab (HR = 0.34, 95% CI: 0.10–1.18, P = 0.088).

**Conclusion:**

Compared to SS, MFR was associated with improved outcomes of subsequent systemic therapy in patients with spinal metastases from NSCLC, albeit at the cost of increased surgical trauma and a higher risk of intraoperative complications. In addition, MFR was associated primarily with disease stabilization rather than objective tumor regression. Notably, the combination of MFR with denosumab was associated with longer overall survival. These findings support the consideration of MFR in carefully selected patients, particularly when optimizing the response to systemic therapy is a key clinical objective. Importantly, given the small sample size, the observed differences in disease control rate and subgroup survival should be interpreted as hypothesis-generating.

**Level of evidence:**

Level IV

**Supplementary Information:**

The online version contains supplementary material available at 10.1186/s12957-026-04300-y.

## Key points

1. MFR was associated with improved disease control compared to separation surgery in NSCLC spinal metastases.

2. MFR was associated with greater surgical trauma and higher complication rates.

3. No significant OS difference was found overall between surgical strategies.

4. MFR combined with denosumab was associated with significantly longer overall survival in subgroup analysis.

**Supplementary Information:**

The online version contains supplementary material available at 10.1186/s12957-026-04300-y.

## Introduction

Lung cancer is the most commonly diagnosed cancer and the leading cause of death from cancer across the world [1, 2]. Bone is one of the most common sites of metastases in advanced lung cancer, among which spinal metastasis is the most frequent, which occurs in about 30%–36% of lung cancer patients [3, 4]. Spinal metastases often lead to pain, pathological fractures, neurological deficits, and significant decline in quality of life [5].

With the advent of immune checkpoint inhibitors (ICIs), the systemic treatment paradigm for non-small cell lung cancer (NSCLC) has undergone a major transformation [6]. ICIs targeting PD-1/PD-L1 have significantly improved survival outcomes in selected patients, including those with advanced disease [7]. However, accumulating evidence suggests that the presence of bone metastases may compromise the responsiveness of the primary tumor to systemic therapy, particularly immunotherapy [8–10]. Whether surgical strategies for spinal metastases—by reducing varying degrees of metastatic tumor burden—can influence the efficacy of subsequent systemic therapy remains unclear.

Even though the paradigm of surgical treatment for spinal metastases has gradually shifted to less invasive procedures to facilitate stereotactic body radiotherapy (SBRT), maximal feasible resection (MFR) is still regularly performed in selected patients. Therefore, in this retrospective cohort study, we compared systemic therapy outcomes between patients who underwent separation surgery (SS) and those who received MFR for spinal metastases from NSCLC.

## Materials and methods

### Study design and setting

**Fig. 1:**
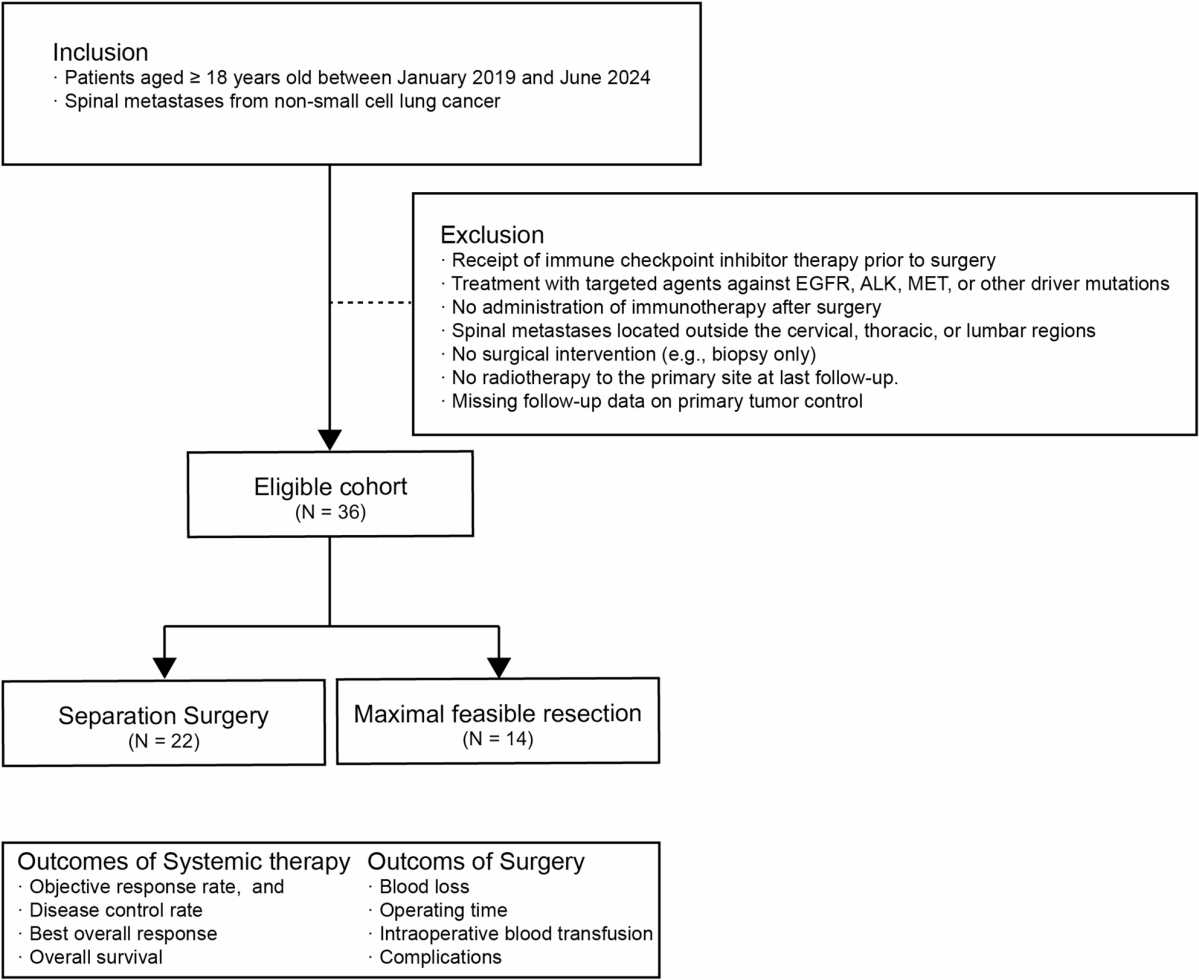
Flow diagram of patient selection and clinical outcomes

Adult patients (≥ 18 years) with spinal metastases from NSCLC who underwent separation surgery or maximal feasible resection at our institution between January 2019 and June 2024 were retrospectively identified based on predefined inclusion and exclusion criteria (Fig. 1). A total of 36 patients were included in the final analysis, of whom 22 underwent SS and 14 underwent MFR. Ethical approval was waived by the Medical Ethics Committee of Shanghai Changzheng Hospital, as it involved only retrospective analysis of anonymized data. The Strengthening the Reporting of Observational Studies in Epidemiology (STROBE) checklist for cohort studies was adhered to in the preparation of this manuscript (Supplemental Digital Content 1) [11].

### Definition of surgical strategies

The SS refers to a posterolateral epidural decompression with posterior instrumented fusion that focused on reconstitution of spinal fluid space to create a 2-mm margin between the tumor and spinal cord, but without resection of the vertebral body or paraspinal tumor [12].

MFR is an umbrella term encompassing both subtotal corpectomy (tumor curettage/debulking) and total spondylectomy [13]. Unlike separation surgery, which is primarily aimed at decompression, MFR for spinal metastases integrates both neural decompression and tumor cytoreduction. The extent of cytoreduction is determined by factors such as the surgeon’s experience, the availability of surgical equipment, and the patient’s physiological tolerance; nevertheless, all such procedures fall within the definition of MFR [13]. Importantly, MFR represents a surgical strategy rather than a standardized intervention, and its current definition carries limited external generalizability, warranting further refinement and subclassification in future studies.

The choice of surgical approach was determined through a shared decision-making process involving the patient, spine surgeon, and oncologic team. Postoperative care was individualized based on disease severity, with no systematic differences in management between the two surgical strategies. As immunotherapy is generally not associated with impaired wound healing, it was resumed in the second postoperative week.

### Outcome measures

Systemic therapy outcomes included the objective response rate (ORR), disease control rate (DCR), and best overall response (BOR). All responses were evaluated at 3 months after treatment initiation according to the Response Evaluation Criteria in Solid Tumors (RECIST) version 1.1. The ORR was defined as the proportion of patients whose best overall response was either a confirmed complete response (CR) or partial response (PR), as determined by blinded independent central review (BICR) using RECIST version 1.1. The DCR included patients who achieved a confirmed CR, PR, or stable disease (SD) as their best overall response, also assessed by BICR per RECIST version 1.1. The best overall BOR was defined as the best response recorded between the date of surgery and the earliest of either documented disease progression or initiation of subsequent anticancer therapy.

Surgical outcomes encompassed intraoperative blood loss, operative time, intraoperative blood transfusion, and postoperative complications. Overall survival was also assessed as a key clinical endpoint.

### Explanatory variables

Explanatory variables included demographic characteristics (age and gender) and preoperative functional assessments, including the Eastern Cooperative Oncology Group (ECOG) performance status [14] and the Frankel grade [15]. Neurological status was evaluated using the Epidural Spinal Cord Compression (ESCC) scale [16], and prognostic assessment was conducted based on the revised Tokuhashi score [17]. Spinal stability was assessed using the Spinal Instability Neoplastic Score (SINS) [18].

Other disease-related variables included the anatomical location of spinal metastases, histologic subtype, presence of solitary bone metastasis, visceral involvement, and the interval between initial diagnosis and bone metastasis. Treatment-related factors included prior radiation to the index spinal lesion, use of bone-modifying agents, and receipt of postoperative chemotherapy.

All variables were retrospectively extracted from electronic medical records by independent investigators who were blinded to the clinical outcomes.

### Statistical analysis

Categorical variables were presented as counts and percentages (%), while continuous variables were reported as medians with interquartile ranges (IQRs), due to their non-normal distribution. Nonparametric tests were applied to compare baseline characteristics and outcomes, including the Mann–Whitney U test for continuous variables and Fisher’s exact test for categorical variables. Baseline variables with a p-value < 0.20 in univariable analysis were entered into the multivariable logistic regression model. The proportional hazards assumption was assessed using Schoenfeld residuals, and no violation was detected. A two-tailed P-value of < 0.05 was considered statistically significant. All analyses were conducted using R software (version 4.3.1).

## Results

### Study population

A total of 36 patients were included in the study, with a median follow-up time of 337 days (IQR: 292.75–549.75 days). The median age was 63 years (IQR: 53.8–67.2), and 30 patients (83.3%) were male. Most patients had good preoperative performance status, with 86.1% presenting an ECOG score of 0–2 and 86.1% classified as Frankel Grade D–E. Based on the revised Tokuhashi score, 61.1% of patients were categorized as 0–8, indicating a relatively limited prognosis. Neurological compression was evaluated using the ESCC scale, with the majority of patients having grade 2 or 3 compression. Regarding spinal stability, 47.2% were considered potentially unstable, while 27.8% were classified as unstable based on the SINS category. The most commonly involved spinal levels were the lumbar (41.7%) and thoracic (38.9%) regions. The median interval from initial diagnosis to bone metastasis was 2 months (IQR: 1.00–3.00). Adenocarcinoma accounted for the majority of histologic subtypes (77.8%). Solitary bone metastasis and visceral metastasis were present in 25.0% and 30.6% of patients, respectively. A small proportion had received previous radiation to the index lesion (11.1%). Regarding bone-modifying agents, 58.3% received denosumab, while 30.6% were treated with bisphosphonates. Postoperative chemotherapy was administered in 83.3% of cases (Table 1).

**Table 1:** Baseline Characteristics of Patients Undergoing SS (*n* = 22) vs. MFR (*n* = 14) for Spinal Metastases

	All	SS	MFR	*P* value
	*N* = 36	*N* = 22	*N* = 14	
Median age, year (IQR)	63.0 [53.8;67.2]	63.5 [56.0;67.0]	61.5 [48.2;68.5]	0.526
Gender, n(%): Male: Female	6 (16.7%)	5 (22.7%)	1 (7.14%)	0.370
Preoperative ECOG score, n (%):				0.628
Grade 0–2	31 (86.1%)	18 (81.8%)	13 (92.9%)	
Grade 3–4	5 (13.9%)	4 (18.2%)	1 (7.14%)	
Preoperative Frankel, n (%):				1.000
Grade A-C	5 (13.9%)	3 (13.6%)	2 (14.3%)	
Grade D-E	31 (86.1%)	19 (86.4%)	12 (85.7%)	
Revised Tokuhashi score, n (%):				0.867
Score 0–8	22 (61.1%)	13 (59.1%)	9 (64.3%)	
Score 9–11	12 (33.3%)	8 (36.4%)	4 (28.6%)	
Score 12–15	2 (5.56%)	1 (4.55%)	1 (7.14%)	
ESCC score, n (%):				0.926
0-1b	4 (11.1%)	2 (9.09%)	2 (14.3%)	
1c	8 (22.2%)	5 (22.7%)	3 (21.4%)	
2	10 (27.8%)	7 (31.8%)	3 (21.4%)	
3	14 (38.9%)	8 (36.4%)	6 (42.9%)	
SINS category, n (%):				0.511
Stable	9 (25.0%)	7 (31.8%)	2 (14.3%)	
Potentially unstable	17 (47.2%)	9 (40.9%)	8 (57.1%)	
Unstable	10 (27.8%)	6 (27.3%)	4 (28.6%)	
Index spinal level, n (%):				0.530
Cervical	5 (13.9%)	2 (9.09%)	3 (21.4%)	
Thoracic	14 (38.9%)	8 (36.4%)	6 (42.9%)	
Lumbar	15 (41.7%)	10 (45.5%)	5 (35.7%)	
Combined	2 (5.56%)	2 (9.09%)	0 (0.00%)	
Interval to bone metastasis, month (IQR)	2.00 [1.00;3.00]	2.00 [1.00;3.00]	2.50 [2.00;3.00]	0.430
Pathology, n (%):				0.441
Adenocarcinoma	28 (77.8%)	16 (72.7%)	12 (85.7%)	
Squamous carcinoma	8 (22.2%)	6 (27.3%)	2 (14.3%)	
Solitary bone metastasis, n (%): Yes	9 (25.0%)	3 (13.6%)	6 (42.9%)	0.111
Visceral metastasis, n (%): Yes	11 (30.6%)	9 (40.9%)	2 (14.3%)	0.142
Previously radiated, n (%): Yes	4 (11.1%)	2 (9.09%)	2 (14.3%)	0.634
Bone-Modifying Agents, n (%):				0.262
No	4 (11.1%)	2 (9.09%)	2 (14.3%)	
Bisphosphonate	11 (30.6%)	9 (40.9%)	2 (14.3%)	
Denosumab	21 (58.3%)	11 (50.0%)	10 (71.4%)	
Chemotherapy After Surgery, n (%): Yes	30 (83.3%)	18 (81.8%)	12 (85.7%)	1.000

There were no significant differences in baseline characteristics between the two surgical groups (all *P* > 0.05; Table 1; Supplementary Table 1).

### Systemic therapy outcomes

Systemic therapy outcomes differed between the SS group and the MFR group. The ORR was higher in the MFR group (21.4%) compared to the SS group (4.55%), although the difference did not reach statistical significance (*P* = 0.277). Notably, the DCR was significantly greater in the MFR group (71.4%) than in the SS group (27.3%) (*P* = 0.024). Analysis of the BOR showed a trend favoring the MFR group (*P* = 0.082). In detail, partial response (PR) was observed in 21.4% of patients in the MFR group versus 4.55% in the SS group. Stable disease (SD) accounted for 71.4% in the MFR group and 63.6% in the SS group, while progressive disease (PD) occurred less frequently in the MFR group (7.14%) compared to the SS group (31.8%) (Table 2).

**Table 2:** Systemic Therapy Outcomes of Patients Undergoing SS (*n* = 22) vs. MFR (*n* = 14) for Spinal Metastases

	SS	MFR	*P* value
	*N* = 22	*N* = 14	
Objective response rate, n (%)	1 (4.55%)	3 (21.4%)	0.277
Disease control rate, n (%)	6 (27.3%)	10 (71.4%)	0.024
Best overall response, n (%):			0.082
PR	1 (4.55%)	3 (21.4%)	
SD	14 (63.6%)	10 (71.4%)	
PD	7 (31.8%)	1 (7.14%)	

### Surgical outcomes

The median estimated intraoperative blood loss was significantly higher in the MFR group (800 mL [IQR: 650–1425]) than in the SS group (500 mL [IQR: 100–1000]) (*P* = 0.041). Similarly, intraoperative blood transfusion was required more frequently in the MFR group (71.4%) compared to the SS group (31.8%) (*P* = 0.048). The median operating time tended to be longer in the MFR group (242 min [IQR: 186–295]) than in the SS group (172 min [IQR: 91.2–255]), though this difference was not statistically significant (*P* = 0.115). Intraoperative complications occurred more frequently in the MFR group (*P* = 0.017), including two cases of dural tear and two cases of massive bleeding. No intraoperative complications were observed in the SS group. Postoperative complications were observed in 14.3% of patients in the MFR group and in 4.55% of patients in the SS group, without a statistically significant difference (*P* = 0.547) (Table 3).

**Table 3:** Intraoperative and Postoperative Outcomes of Patients Undergoing SS (*n* = 22) vs. MFR (*n* = 14) for Spinal Metastases

	SS	MFR	*P* value
	*N* = 22	*N* = 14	
Median estimated blood loss, ml (IQR)	500 [100;1000]	800 [650;1425]	0.041
Intraoperative blood transfusion, n (%): Yes	7 (31.8%)	10 (71.4%)	0.048
Median operating time, min (IQR)	172 [91.2;255]	242 [186;295]	0.115
Intraoperative complications, n (%):			0.017
Dural tear	0 (0.00%)	2 (14.3%)	
Massive bleeding	0 (0.00%)	2 (14.3%)	
No	22 (100%)	10 (71.4%)	
Postoperative complications, n (%): Yes	1 (4.55%)	2 (14.3%)	0.547

### Survival outcomes

**Fig. 2:**
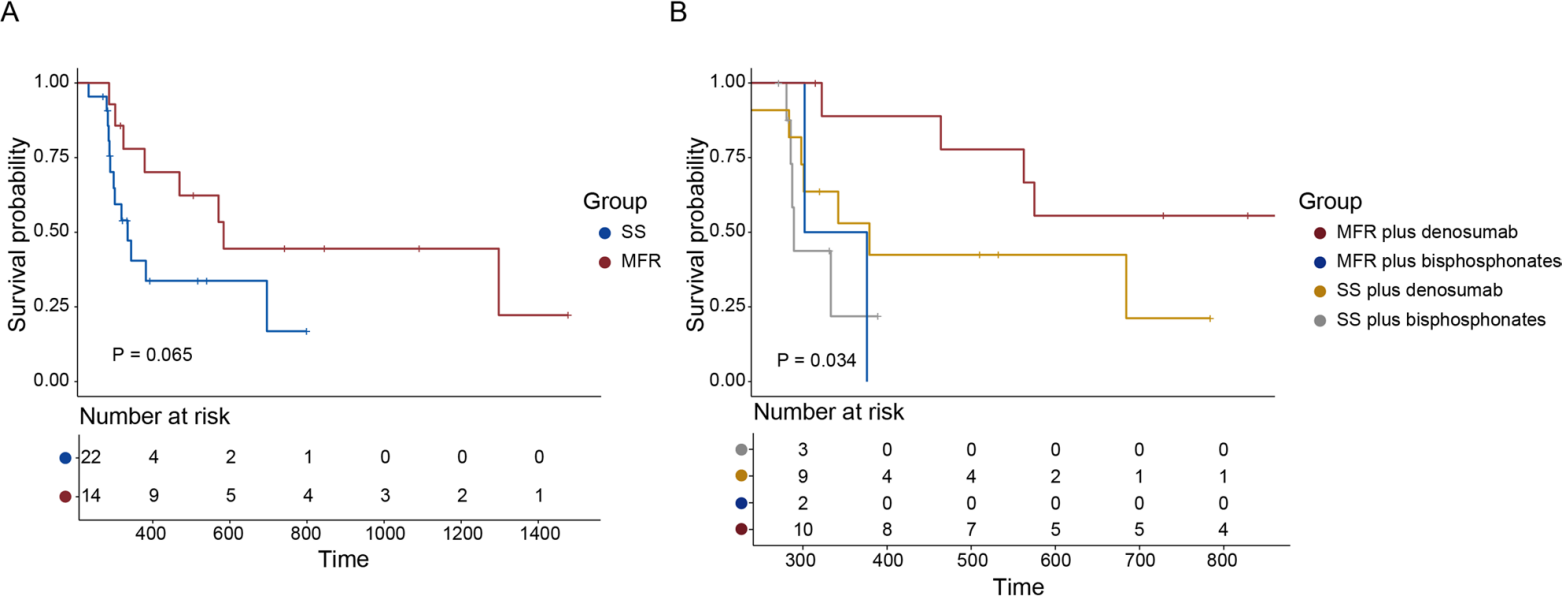
Kaplan–Meier curves of overall survival stratified by surgical strategy and bone-modifying agent use. **A** Overall survival in patients undergoing MFR versus SS. **B** Subgroup analysis based on surgical strategy and type of bone-modifying agents

There was no statistically significant difference in overall survival (OS) between patients who underwent maximal feasible resection (MFR) and those who received separation surgery (SS). The median OS in the MFR group was 23.7 months (95% CI: 18.8–29.4), compared to 20.6 months (95% CI: 17.5–22.5) in the SS group, with a hazard ratio (HR) of 0.42 (95% CI: 0.16–1.08, *P* = 0.065). No statistically significant difference in overall survival was observed between groups (Fig. 2A). These findings should be interpreted as exploratory given the limited sample size.

To further explore the potential interaction between surgical strategy and bone-modifying agents (BMAs), subgroup analysis was conducted. Notably, patients who received MFR combined with denosumab were associated with significantly prolonged OS compared to those treated with SS plus bisphosphonates (HR = 0.14, 95% CI: 0.03–0.61, *P* = 0.009) and MFR plus bisphosphonates (HR = 0.15, 95% CI: 0.02–0.95, *P* = 0.044). In addition, a favorable trend toward improved OS was observed when comparing MFR plus denosumab with SS plus denosumab (HR = 0.34, 95% CI: 0.10–1.18, *P* = 0.088), although this did not reach statistical significance (Fig. 2B).

## Discussion

To our knowledge, this is the first study to make a direct comparison of systemic therapy response between these two surgical modalities. In this retrospective cohort, we observed that MFR, although not associated with a higher objective response rate, was linked to improved disease control compared to separation surgery in patients with spinal metastases from NSCLC. However, this benefit was accompanied by increased surgical trauma and a higher rate of intraoperative complications. While overall survival did not differ significantly between the two surgical strategies in the overall cohort, subgroup analysis suggested that MFR combined with denosumab may be associated with improved survival outcomes. These findings indicate that, in selected patients, more extensive surgical resection combined with bone-modifying agents could provide additional clinical benefit.

Previous studies have demonstrated that bone metastasis may negatively impact the efficacy of ICIs in patients with NSCLC. Zhang et al. [9]reported that the presence of bone metastasesis associated with poorer clinical outcomes following ICI treatment, potentially due to the “cold” tumor immune microenvironment within bone lesions. Similarly, Zhu et al. [8]systematically investigated the influence of bone metastasis on ICI response across pan-cancer cohorts and murine models. Their findings revealed that osseous metastases can induce resistance to ICIs even in extraosseous tumors, mediated by osteoclast-derived osteopontin (OPN), which impairs CD8⁺ T cell differentiation and recruitment. Importantly, targeting osteoclastogenesis via receptor activator of nuclear factor kappa-B ligand (RANKL) blockade or OPN neutralization was shown to restore ICI responsiveness in preclinical models and clinical cohorts.

Consistent with these findings, our study also observed an association between bone metastatic burden and reduced disease control rate. However, unlike previous studies that primarily focused on the presence or absence of bone metastases, our investigation is the first to directly compare systemic therapy outcomes based on different surgical strategies for spinal metastases. This distinction underscores the potential impact of surgical approach selection in modulating systemic therapeutic efficacy, particularly when combined with bone-modifying agents such as denosumab. Although direct mechanistic evidence was not investigated in this study, one potential explanation is that surgical cytoreduction may reduce tumor burden within the bone marrow microenvironment, potentially mitigating local immunosuppressive signaling and thereby supporting systemic immune responsiveness. Bone metastases have been reported to alter hematopoietic and immune cell composition, potentially contributing to systemic immune dysfunction. Reduction of tumor volume may partially restore immune homeostasis and enhance sensitivity to systemic therapy. However, this hypothesis remains speculative and warrants further mechanistic investigation. Importantly, given the small sample size, the observed differences in disease control rate and subgroup survival should be interpreted as hypothesis-generating.

Similar to our findings, studies by Cao et al. [19] and Tobert et al. [20] reported that patients undergoing separation surgery experienced lower intraoperative blood loss and shorter operative times compared to more extensive surgical procedures. In our cohort, these technical advantages were accompanied by a lower rate of intraoperative complications in the separation surgery group, whereas patients undergoing maximal feasible resection exhibited a higher incidence of postoperative complications, likely reflecting the increased surgical complexity and trauma associated with more aggressive resections.

### Limitation

This study had a number of limitations. First, the retrospective nature of our study introduces an inherent risk of selection and confounding bias, particularly in the choice of surgical approach. Patients undergoing MFR were more likely to present with limited metastatic burden, whereas those treated with separation surgery more frequently exhibited visceral involvement. Such differences in baseline disease distribution may have influenced systemic treatment response and survival outcomes independent of the surgical strategy itself. Second, we were unable to accurately quantify the burden of bone and extraosseous metastases, limiting our ability to fully adjust for disease severity. Third, due to real-world clinical practice, most patients received chemotherapy in combination with immunotherapy, which may have diluted the specific contribution of immune checkpoint blockade and reflected the overall response to systemic therapy rather than immunotherapy alone. Fourth, our study lacks mechanistic validation at the experimental level. Future studies with larger sample sizes and prospective designs are warranted to determine whether the observed differences are statistically and clinically significant.

## Conclusion

In this retrospective cohort, MFR was associated with improved disease control and was accompanied by greater surgical trauma and a higher complication rate compared to separation surgery. While overall survival did not differ significantly between the two groups, subgroup analysis indicated that MFR combined with denosumab may confer a survival advantage. These findings suggest that, in carefully selected patients, MFR in combination with bone-modifying agents may offer additional clinical benefit, and warrant further validation in prospective studies. 

## Supplementary Information

Supplementary Material 1.

Supplementary Material 2.

## Data Availability

The data generated during the current study are available from the corresponding author upon reasonable request.

## References

[1] Siegel RL, Giaquinto AN, Jemal A. Cancer statistics, 2024. CA Cancer J Clin. 2024;74(1):12–49. 38230766 10.3322/caac.21820

[2] Han B, et al. Cancer incidence and mortality in China, 2022. J Natl Cancer Cent. 2024;4(1):47–53. 39036382 10.1016/j.jncc.2024.01.006 PMC11256708

[3] Rief H, et al. The stability of osseous metastases of the spine in lung cancer–a retrospective analysis of 338 cases. Radiat Oncol. 2013;8(1):200. 23937907 10.1186/1748-717X-8-200 PMC3751223

[4] Zheng J, et al. Prognostic factors and outcomes of surgical intervention for patients with spinal metastases secondary to lung cancer: an update systematic review and meta analysis. Eur Spine J. 2023;32(1):228–43. 36372842 10.1007/s00586-022-07444-z PMC9660217

[5] Chiu RG, Mehta AI. Spinal Metastases Jama. 2020;323(23):2438. 32543685 10.1001/jama.2020.0716

[6] Riely GJ, et al. Non-Small Cell Lung Cancer, Version 4.2024, NCCN Clinical Practice Guidelines in Oncology. J Natl Compr Canc Netw. 2024;22(4):249–74. 38754467 10.6004/jnccn.2204.0023

[7] Reck M, et al. Pembrolizumab versus Chemotherapy for PD-L1-Positive Non-Small-Cell Lung Cancer. N Engl J Med. 2016;375(19):1823–33. 27718847 10.1056/NEJMoa1606774

[8] Cheng JN et al. Bone metastases diminish extraosseous response to checkpoint blockade immunotherapy through osteopontin-producing osteoclasts. Cancer Cell. 2025;43(6):1093–1107.e9. 10.1016/j.ccell.2025.03.036 40280123

[9] Zhu YJ, et al. Bone metastasis attenuates efficacy of immune checkpoint inhibitors and displays cold immune characteristics in Non-small cell lung cancer. Lung Cancer. 2022;166:189–96. 35306320 10.1016/j.lungcan.2022.03.006

[10] Qiang H, et al. Pembrolizumab monotherapy or combination therapy for bone metastases in advanced non-small cell lung cancer: a real-world retrospective study. Transl Lung Cancer Res. 2022;11(1):87–99. 35242630 10.21037/tlcr-21-1033 PMC8825649

[11] von Elm E, et al. Strengthening the Reporting of Observational Studies in Epidemiology (STROBE) statement: guidelines for reporting observational studies. BMJ. 2007;335(7624):806–8. 17947786 10.1136/bmj.39335.541782.AD PMC2034723

[12] Barzilai O, et al. Integrating Evidence-Based Medicine for Treatment of Spinal Metastases Into a Decision Framework: Neurologic, Oncologic, Mechanicals Stability, and Systemic Disease. J Clin Oncol. 2017;35(21):2419–27. 28640703 10.1200/JCO.2017.72.7362

[13] Shen ZZ, et al. Maximal feasible resection versus hybrid therapy in spinal metastases: an updated narrative review. World J Surg Oncol. 2025;23(1):357. 41039525 10.1186/s12957-025-04009-4 PMC12492667

[14] Oken MM, et al. Toxicity and response criteria of the Eastern Cooperative Oncology Group. Am J Clin Oncol. 1982;5(6):649–55. 7165009

[15] van Middendorp JJ, et al. Diagnosis and prognosis of traumatic spinal cord injury. Global Spine J. 2011;1(1):1–8. 24353930 10.1055/s-0031-1296049 PMC3864437

[16] Bilsky MH, et al. Reliability analysis of the epidural spinal cord compression scale. J Neurosurg Spine. 2010;13(3):324–8. 20809724 10.3171/2010.3.SPINE09459

[17] Tokuhashi Y, et al. A revised scoring system for preoperative evaluation of metastatic spine tumor prognosis. Spine (Phila Pa 1976). 2005;30(19):2186–91. 16205345 10.1097/01.brs.0000180401.06919.a5

[18] Fisher CG, et al. A novel classification system for spinal instability in neoplastic disease: an evidence-based approach and expert consensus from the Spine Oncology Study Group. Spine (Phila Pa 1976). 2010;35(22):E1221–9. 20562730 10.1097/BRS.0b013e3181e16ae2

[19] Cao S, et al. A comparison of two different surgical procedures in the treatment of isolated spinal metastasis patients with metastatic spinal cord compression: a case-control study. Eur Spine J. 2022;31(6):1583–9. 34668050 10.1007/s00586-021-07032-7

[20] Amelink J, et al. Comparative Analysis of Surgical Outcomes in Separation Surgery Versus Anterior Reconstruction for Metastatic Epidural Spinal Cord Compression. Spine (Phila Pa 1976). 2025;50(9):612–9. 39523679 10.1097/BRS.0000000000005207

