# Supplementary material for: Systemic therapy outcomes following separation surgery versus maximal feasible resection for spinal metastases from non-small cell lung cancer: a real-world retrospective cohort study
Source: World J Surg Oncol. 2026 Mar 11;24:177. doi: 10.1186/s12957-026-04300-y (PMC13088601; doi:10.1186/s12957-026-04300-y)
Supplement: Supplementary file 1 — Supplementary Material 1. [file 12957_2026_4300_MOESM1_ESM.docx]

STROBE (STrengthening the Reporting of OBservational studies in Epidemiology) Checklist

For this purpose a cohort study (the term used by STROBE) is considered a longitudinal study typically reporting outcomes of treatment in one or more cohorts; a case-control study is one identifying factors in outcomes; a cross-sectional study is one to identify the prevalence of factors or characteristics in a population at a single point in time.

This table is modified from and used with the permission of The STROBE Initiative, [www.strobe-statement.org](http://www.strobe-statement.org).

STROBE Statement—Checklist of items that should be included in reports of ***cohort studies***

|  | Item No | Recommendation | Please insert check where included or N/A where not applicable |
| --- | --- | --- | --- |
| **Title and abstract** | 1 | (*a*) Indicate the study’s design with a commonly used term in the title or the abstract | 2 |
|  |  | (*b*) Provide in the abstract an informative and balanced summary of what was done and what was found | 2 |
| Introduction | | |  |
| Background/rationale | 2 | Explain the scientific background and rationale for the investigation being reported | 3 |
| Objectives | 3 | State specific objectives, including any prespecified hypotheses | 3 |
| Methods | | |  |
| Study design | 4 | Present key elements of study design early in the paper | 3 |
| Setting | 5 | Describe the setting, locations, and relevant dates, including periods of recruitment, treatment, follow-up, and data collection | 4 |
| Participants | 6 | (*a*) *Cohort study*—Give the eligibility criteria, and the sources and methods of selection of participants. Describe methods of follow-up  *Case-control study*—Give the eligibility criteria, and the sources and methods of case ascertainment and control selection. Give the rationale for the choice of cases and controls  *Cross-sectional study*—Give the eligibility criteria, and the sources and methods of selection of participants | Flow diagram |
|  |  | (*b*) *Cohort study*—For matched studies, give matching criteria and number of treated and untreated  *Case-control study*—For matched studies, give matching criteria and the number of controls per case | Flow diagram |

| Participants | 13* | (a) Report numbers of individuals at each stage of study—eg numbers potentially eligible, examined for eligibility, confirmed eligible, included in the study, completing follow-up, and analysed | 3 |
| --- | --- | --- | --- |
|  |  | (b) Give reasons for non-participation at each stage | NA |
| Descriptive data | 14* | (a) Give characteristics of study participants (eg demographic, clinical, social) and information on other treatments and potential confounders | 5 |
|  |  | (b) Indicate number of participants with missing data for each variable of interest | NA |
|  |  | (c) *Cohort study*—Summarise follow-up time (eg, average and total amount) | 5 |
| Variables | 7 | Clearly define all outcomes, treatments, predictors, potential confounders, and effect modifiers. Give diagnostic criteria, if applicable | 5 |
| Data sources/ measurement | 8* | For each variable of interest, give sources of data and details of methods of assessment (measurement). Describe comparability of assessment methods if there is more than one group | *4* |
| Bias | 9 | Describe any efforts to address potential sources of bias | 5 |
| Study size | 10 | Explain how the study size was arrived at | 5 |
| Quantitative variables | 11 | Explain how quantitative variables were handled in the analyses. If applicable, describe which groupings were chosen and why | 5 |
| Statistical methods | 12 | (*a*) Describe all statistical methods, including those used to control for confounding | 5 |
|  |  | (*b*) Describe any methods used to examine subgroups and interactions | 5 |
|  |  | (*c*) Explain how missing data were addressed | NA |
|  |  | (*d*) *Cohort study*—If applicable, explain how loss to follow-up was addressed  *Case-control study*—If applicable, explain how matching of cases and controls was addressed  *Cross-sectional study*—If applicable, describe analytical methods taking account of sampling strategy | NA |
|  |  | (*e*) Describe any sensitivity analyses | NA |

*Give information separately for cases and controls.

**Note:** An Explanation and Elaboration article discusses each checklist item and gives methodological background and published examples of transparent reporting. The STROBE checklist is best used in conjunction with this article (freely available on the Web sites of PLoS Medicine at http://www.plosmedicine.org/, Annals of Internal Medicine at http://www.annals.org/, and Epidemiology at http://www.epidem.com/). Information on the STROBE Initiative is available at http://www.strobe-statement.org.

Modifications to STROBE Guidelines: We added a fourth column for authors to check inclusion. You must include all items in your manuscript unless the information is not applicable. Information on the study cohort (Items 13 and 14 in the STROBE guidelines) should be provided in Patients and Methods, not in Results; we have omitted the portions of the STROBE guidelines related to Results and Discussion (see our guidelines). The STROBE guidelines were developed for epidemiological studies; “exposed” or “exposure” have been modified with the words “treated” or “treatment.”
