# Supplementary material for: Systemic therapy outcomes following separation surgery versus maximal feasible resection for spinal metastases from non-small cell lung cancer: a real-world retrospective cohort study
Source: World J Surg Oncol. 2026 Mar 11;24:177. doi: 10.1186/s12957-026-04300-y (PMC13088601; doi:10.1186/s12957-026-04300-y)
Supplement: Supplementary file 2 — Supplementary Material 2. [file 12957_2026_4300_MOESM2_ESM.docx]

Supplementary Table 1. Multivariable Logistic Regression Analysis of Factors Associated with Surgical Strategy (MFR vs. SS)

|  | **All** | **SS** | **MFR** | **P value** |
| --- | --- | --- | --- | --- |
|  | ***N=36*** | ***N=22*** | ***N=14*** |  |
| Solitary bone metastasis, n (%): Yes | 9 (25.0%) | 3 (13.6%) | 6 (42.9%) | 0.075 |
| Visceral metastasis, n (%): Yes | 11 (30.6%) | 9 (40.9%) | 2 (14.3%) | 0.132 |
